# Distinct soil bacterial communities along a small-scale elevational gradient in alpine tundra

**DOI:** 10.3389/fmicb.2015.00582

**Published:** 2015-06-09

**Authors:** Congcong Shen, Yingying Ni, Wenju Liang, Jianjun Wang, Haiyan Chu

**Affiliations:** ^1^State Key Laboratory of Soil and Sustainable Agriculture, Institute of Soil Science, Chinese Academy of Sciences, NanjingChina; ^2^University of Chinese Academy of Sciences, BeijingChina; ^3^State Key Laboratory of Forest and Soil Ecology, Institute of Applied Ecology, Chinese Academy of Sciences, ShenyangChina; ^4^State Key Laboratory of Lake Science and Environment, Institute of Geography and Limnology, Chinese Academy of Sciences, NanjingChina

**Keywords:** Changbai Mountain tundra, elevation, soil carbon and nitrogen contents, soil bacterial community, phylogenetic relatedness, pyrosequencing

## Abstract

The elevational diversity pattern for microorganisms has received great attention recently but is still understudied, and phylogenetic relatedness is rarely studied for microbial elevational distributions. Using a bar-coded pyrosequencing technique, we examined the biodiversity patterns for soil bacterial communities of tundra ecosystem along 2000–2500 m elevations on Changbai Mountain in China. Bacterial taxonomic richness displayed a linear decreasing trend with increasing elevation. Phylogenetic diversity and mean nearest taxon distance (MNTD) exhibited a unimodal pattern with elevation. Bacterial communities were more phylogenetically clustered than expected by chance at all elevations based on the standardized effect size of MNTD metric. The bacterial communities differed dramatically among elevations, and the community composition was significantly correlated with soil total carbon (TC), total nitrogen, C:N ratio, and dissolved organic carbon. Multiple ordinary least squares regression analysis showed that the observed biodiversity patterns strongly correlated with soil TC and C:N ratio. Taken together, this is the first time that a significant bacterial diversity pattern has been observed across a small-scale elevational gradient. Our results indicated that soil carbon and nitrogen contents were the critical environmental factors affecting bacterial elevational distribution in Changbai Mountain tundra. This suggested that ecological niche-based environmental filtering processes related to soil carbon and nitrogen contents could play a dominant role in structuring bacterial communities along the elevational gradient.

## Introduction

Mountainsides often provide a natural laboratory for studies of biodiversity and biogeography (Lomolino, 2001; Rahbek, 2005). The study of elevational diversity patterns is not only indispensable to a comprehensive understanding of basic ecology, but can also provide evidence for predicting the influence of climate change on ecosystems. The effect of elevational gradients on plant and animal diversity has been extensively documented over the past century. Studies on the microbial ecology of these environments are rare, and the state of knowledge is generally rudimentary. It is so far unclear whether there is any consistent trend in soil bacterial diversity with elevation in mountainous regions, with a decreasing (Bryant et al., 2008), unimodal (Singh et al., 2012), or inconsistent pattern (Singh et al., 2014) found in previous studies. Other studies found no apparent trend with elevation for bacteria or ammonia-oxidizing bacteria (Zhang et al., 2009; Fierer et al., 2011; Shen et al., 2013; Xu et al., 2014; Yuan et al., 2014). Notably, all of these studies focused on a complete or large-scale elevational gradient, with relatively large elevational intervals and contrasting ecosystems. It is well documented that the scale over which biodiversity is sampled will strongly influence the patterns observed (Rahbek, 2005; Green and Bohannan, 2006). Yet there has been no research addressing microbial diversity and community composition of a consistent ecosystem within a small-scale elevational gradient.

Since the development of community phylogenetics, researchers are increasingly using phylogenetic framework to study the forces underlying biodiversity and biogeography patterns (Webb, 2000; Bryant et al., 2008; Vamosi et al., 2009; Jones and Hallin, 2010). This framework mainly focuses on the patterns of phylogenetic relatedness within communities to infer the importance of different ecological and evolutionary processes that organize these communities (Webb et al., 2002; Kembel and Hubbell, 2006; Kembel, 2009). Recently, the study of community phylogenetic structures has been addressed along elevational gradients, but with limited organisms including plants (Bryant et al., 2008; Kluge and Kessler, 2010), hummingbirds (Graham et al., 2009), ants (Machac et al., 2011), and bees (Hoiss et al., 2012). In one of these studies, Hoiss et al. (2012) found a linear decline in species richness but increasing phylogenetic clustering in communities with increasing elevation and concluded that elevation acts as an environmental filter on phylogenetic composition, traits, and diversity in bee communities. To our knowledge, there are only two elevational studies that have tested patterns of phylogenetic relatedness of microbes along an elevation gradient, with one focused on soil Acidobacteria and another on bacteria in a stony stream (Bryant et al., 2008; Wang et al., 2012). Consistently, the studies both concluded that environmental filtering processes were likely to be a prominent force structuring bacterial communities along elevational gradients.

Bacterial consortia display spatial patterns linked to geographic distance (Cho and Tiedje, 2000; Green and Bohannan, 2006; Martiny et al., 2006), soil characteristics (Fierer and Jackson, 2006; Lauber et al., 2009; Chu et al., 2010; Liu et al., 2014), and vegetation type (Knelman et al., 2012; Shen et al., 2013; Zhang et al., 2014). Chu et al. (2010) found that geographic distance was less important than soil pH in driving bacterial latitudinal distribution in Arctic tundra. Other studies have also documented the influence of soil depth and vegetation type on bacterial communities in Arctic and subarctic tundra (Männistö et al., 2007; Chu et al., 2011; Kim et al., 2014; Shi et al., 2015). Since tundra soils are widely recognized as highly nutrient limited, large bodies of studies concentrated on assessing the importance of nutrient availability on microbial diversity and community composition (Nemergut et al., 2008; Sistla et al., 2013; Koyama et al., 2014; Stark et al., 2014). However, much of our understanding of the role of carbon and nitrogen has been limited to N-addition experiments. For example, Koyama et al. (2014) found that soil bacterial community composition altered significantly with increased nutrient availability in Arctic tundra soils. Nemergut et al. (2008) also concluded that chronic N fertilization induced significant shifts in soil carbon dynamics that corresponded to shifts in microbial community structure and function. Stark et al. (2014) found that nutrient availability and pH jointly constrained microbial extracellular enzyme activities in nutrient-poor alpine tundra soils. These results yield useful insights, but given that N inputs might alter soil pH and plant community composition, these experimental results could lead to more confusion about whether shifts in bacterial diversity and community composition were determined directly or indirectly by nutrient availability (Ramirez et al., 2010).

Changbai Mountain tundra marks the southernmost boundary of alpine tundra on the eastern Eurasian continent. This tundra ecosystem, which is representative of alpine tundra in China, was shaped by Quaternary period glacial retreat (Xu et al., 2004). In this study, we investigated soil bacterial biodiversity along the elevation of 2000–2500 m in Changbai Mountain tundra to address the following questions: (1) if there is a significant trend in bacterial diversity along this small-scale elevational gradient, (2) what environmental factors are closely related to bacterial community composition in tundra soils with similar pH. We further hypothesize that environment filtering processes (abiotic factors) are critical in determining bacterial community assembly. To answer above questions and test the hypothesis, we used pyrosequencing and adopted a multifaceted approach to quantify patterns of taxon richness, phylogenetic diversity and phylogenetic relatedness for the communities, and correlated these with measured environmental variables to reveal potential underlying factors.

## Materials and Methods

### Site Selection and Soil Sampling

A detailed background of Changbai Mountain was previously described by Shen et al. (2013, 2014). Changbai Mountain tundra belt, which shaped by Quaternary period glacial retreat and marks the southernmost occurrence of this ecosystem type on the eastern Eurasian continent, distributes between 1950 and 2650 m on Changbai Mountain (Huang, 1999; Xu et al., 2004; Wu et al., 2007). The landforms mainly include three types: Volcanic landforms, Glacial landforms, Periglacial landforms (Wei et al., 2004). The temperature is low and the precipitation is abundant, which forms a tundra-periglacial climate. Mean annual temperature is -4.8°C and mean annual precipitation is 1154 mm. Changbai Mountain tundra is covered by snow from mid-October to mid-May the next year, about 6 months each year. From late May to mid-August is the plant growth period and from late August to mid-October is the plant mature decay period (Zhang et al., 2010). Many of the plant species there are relicts from the Quaternary glacial period. Based on the measurement of species biomass, dominant species organ biomass and vegetation biomass, the first five species in biomass are *Rhododendron chrysanthum* (Pall.), *Vaccinium uliginosum var. alpinum* (L.), *Vaccinium uliginosum* (L.), *Dryas octopetala* (L.), and *Salix rotundifolia* (L.), which are the dominant species in the alpine tundra ecosystem of Changbai Mountain (Wei et al., 2004; Xu et al., 2004).

A recent study on Changbai Mountain tundra divided tundra vegetation into five vegetation types: felsenmeer alpine tundra vegetation (FA), lithic alpine tundra vegetation (LA), typical alpine tundra vegetation (TA), meadow alpine tundra vegetation (MA), swamp alpine tundra vegetation (SA) (Wu et al., 2007). TA generally contains many kinds of dwarf shrubs: *Dryas octopetala* (L.), *Vaccinium uliginosum* (L.), *Rhododendron chrysanthum* (Pall.), *Rhododendron redowskianum* (Maxim.), etc; and some kinds of herbs: *Carex atrata* (Linn.), *Polygonum viviparum* (L.), etc; as well as mosses: *Racomitrium lanuginosum* (Hedw. Bird.), *Racomitrium canescens* (Hedw. Bird.), etc; lichens: *Cladonia rangiferina* (L.), etc. MA mainly contains various herbs: *Carex laevissima* (Nakai.), *Carex atrata* (Linn.), *Bupleurum euphorbioides* (Nakai.), *Oxytropis anertii* (Nakai.), etc; and also some kinds of dwarf shrubs: *Salix rotundifolia* (L.), *Phyllodoce caerulea* (L.), etc. FA and LA distributing above 2500 m, was excluded from this study as the thickness of soil organic layer is less than 5 cm. SA was also excluded due to its high soil moisture. Soil samples were collected from MA and TA vegetation types from the northern slope of alpine tundra on July 29, 2011. From 2000 to 2500 m, we chose six elevations with an elevational interval of 100 m. At each elevation, soil samples were collected from 4 plots (10 m × 10 m) as four independent replicates. In each plot, samples of the soil organic layer (∼10 cm × 10 cm in area) were collected at six random points using a sterile blade and composited together as a single sample. Since both MA and TA has a more than 5 cm thick organic layer, soil samples were sampled to a depth of 0–5 cm directly below the litter layer. Visible roots and residues were removed prior to homogenizing the soil fraction of each sample. The fresh soil samples were sieved through a 2 mm screen and divided into two subsamples. One was stored at 4°C to determine the physical and chemical properties, and the other was stored at -40°C prior to DNA extraction.

### Soil Physicochemical Analyses

Soil pH was measured using a pH meter (FE20-FiveEasy^TM^ pH, Mettler Toledo, Germany) after shaking a soil water (1: 5w/v) suspension for 30 min. Soil moisture was measured gravimetrically. Total carbon (TC) and total nitrogen (TN) contents were measured by elemental analyzer (Vario MAX, Elementar, Germany). Ammonium (NH_4_^+^-N), nitrate (NO_3_^-^-N), dissolved organic carbon (DOC) and dissolved total nitrogen (DTN) were extracted at a ratio of 10 *g* fresh soil to 100 mL 2 M KCl. After shaking for 1 h, NH_4_^+^-N, NO_3_^-^-N, and DTN contents in the filtered extracts were analyzed using a continuous flow analytical system (San^++^ System, Skalar, Holland), and DOC was determined using a TOC analyzer (Multi N/C 3000, Analytik Jena, Germany). Dissolved organic nitrogen (DON) was calculated as follows: DON = DTN – NH_4_^+^–N – NO_3_^-^–N.

### DNA Extraction, Amplification, and Pyrosequencing

Details of DNA extraction, bacterial 16S rRNA genes amplification, and pyrosequencing methods have been described previously (Shen et al., 2013). In brief, soil DNA was extracted using a FastDNA^®^ SPIN Kit for soil (MP Biomedicals, Santa Ana, CA, USA) and an aliquot (50 ng) of purified DNA from each sample was used as a template for amplification. Bacterial 16S rRNA genes were amplified using the primer 515F with the Roche 454 ‘A’ pyrosequencing adapter and a unique 7-bp barcode sequence, while primer 907R contained the Roche 454 ‘B’ sequencing adapter. Polymerase chain reaction (PCR) products were pooled together and purified using an Agarose Gel DNA purification kit (Takara, Otsu, Japan). An equal amount of PCR products from each sample was combined in a single tube to be sequenced on a Roche FLX 454 pyrosequencing machine (454 Life Science, Branford, CT, USA).

### Processing of Pyrosequencing Data

Sequences obtained by pyrosequencing were processed and analyzed following the standard operating procedure described in the website^1^ using Mothur program v.1.27.0 (Schloss et al., 2011). The denoising process was implemented using the shhh.flows command which is the Mothur implementation of the PyroNoise component of the AmpliconNoise suite of programs. Barcode and primer sequences were removed, and sequences shorter than 200 bp with homopolymers longer than 8 bp were removed at the same time. Next, the sequences were aligned against the SILVA-compatible alignment database and then trimmed, so that subsequent analyses were constrained to the same portion of the 16S rRNA gene. Chimeric sequences were detected using the chimera.uchime command that use the sequences as their own reference to run *de novo* detection and identified chimeras were removed after that. The remaining reads were preclustered using the pre-cluster command^2^ to remove erroneous sequences derived from sequencing errors and then clustered using Mothur’s average algorithm. Taxonomic assignment of each OTU (clustered at 97% sequence similarity) was obtained by classifying alignments against Silva reference bacterial taxonomy files using the classify command at 80% Bayesian bootstrap cutoff with 1000 iterations. Sequences were deposited to the MG-RAST metagenomics analysis server^3^ and are available to the public (accession numbers from 4565119.3 to 4565142.3).

For community-level composition and each calculated metric, we accounted for the difference in the sampling efforts among the samples by randomly subsampling 4,900 sequences per sample. The number of sequences for rarefaction was determined according to the sample that yielded the lowest number of sequences after quality filtering (Supplementary Table S3).

### Statistical Analysis

The number of phylotypes (the number of OTUs) was used to estimate the community richness. We chose Faith’s (1992) phylogenetic diversity index values (calculated as the sum of branch lengths between root and tips for a community) to estimate the phylogenetic community diversity.

To determine if the different elevation samples formed unique phylogenetically related clusters, principal co-ordinates analysis (PCoA) of the UniFrac distance matrices were performed. The UniFrac algorithm computes the overall phylogenetic distances (across all taxonomically resolved levels) between all pairs of sample communities in the dataset from neighbor-joining trees using either unweighted (i.e., presence/absence) or weighted (i.e., accounting for taxon relative abundance) data (Lozupone and Knight, 2005). In addition, we tested for significant differences in community composition among elevations using analysis of similarities (ANOSIM) with R statistical software. Canonical correspondence analysis (CCA) was performed to show a visual relationship between environmental factors and bacterial distributions. To further identify the environmental and biogeochemical factors that significantly correlated with community composition we used Mantel tests of Bray–Curtis similarity distance values that were calculated on the presence/absence of the OTUs within each sample using the vegan package of R v.3.1.1 project (R Development Core Team, 2010).

For the phylogenetic community structure, we calculated the mean nearest taxon distance (MNTD) of all of the species pairs occurring in a community based on the observed community dataset (Webb et al., 2002). MNTD is an estimate of the mean phylogenetic relatedness between each OTU in a bacterial community and its nearest relative (Wang et al., 2012). To infer underlying ecological processes with MNTD, the phylogenetic signal in habitat association was tested with Mantel correlograms with 999 randomizations for significance tests (Wang et al., 2013). An environmental-optimum for each OTU was found for each environmental variable as in Stegen et al. (2012). Between-OTU environmental-optimum differences were calculated as Euclidean distances using optima for all the environmental variables. We further calculated the differences in the phylogenetic distances between the observed and randomly generated null communities, and we standardized them using the standardized deviation of phylogenetic distances in 1000 null communities (Webb, 2000). These null communities were generated with the assumption that all species that exist along the elevation are equally able to colonize any elevation without dispersal limitation at local spatial scales, and thus each species has the same expected prevalence (Kembel and Hubbell, 2006; Helmus et al., 2007). The total species richness of each elevation was kept standard, and species at each elevation were chosen randomly without replacement from the pool of species present along the elevation. The obtained standardized effect size measure (ses.MNTD) can be used to test for phylogenetic clustering or overdispersion (Webb, 2000). Negative ses.MNTD values and low quantiles (*P* < 0.05) indicate that co-occurring species are more closely related than expected by chance (clustering), whereas positive values and high quantiles (*P* > 0.95) indicate that the co-occurring species are less closely related than expected by chance (overdispersion; Webb, 2000). These analyses were implemented in the R environment^4^ with the package Picante 1.6-2 (Kembel et al., 2010).

To correlate the observed biodiversity patterns with the environmental variables, we used multiple ordinary least squares (OLS) regression. Before that, strong correlated variables were dereplicated according to their correlation (i.e., one of the two variables was selected if the Pearson correlation is higher than 0.7. Usually we only select the most ecologically related factor from the significant correlated variables). All of the environmental variables and biodiversity metrics were standardized at a mean of 0 and a SD of 1. Akaike’s information criterion was used to identify the most parsimonious model (Fotheringham et al., 2002). The regression analyses were performed in the R environment with the package MASS 7.3–33.

## Results

### Bacterial Community Composition

In total, we obtained 257,229 quality sequences for all soil samples, which ranged from 4931 to 20477 sequences per sample with an average length of approximately 400 bp (Supplementary Table S3). A total of 11961 unique OTUs were identified and were assigned to more than 39 bacterial phyla. Among the identified groups, *Alphaproteobacteria* (26%) were the most abundant across the six elevation gradient soils and *Acidobacteria* were the second most abundant phylum, accounting for 17% of all sequences (**Figure 2A**, Supplementary Table S4). Testing by ANOSIM revealed that OTU-based taxonomic community composition differed significantly among elevations. However, the difference in community composition between 2000 and 2100 m, 2100 and 2200 m, 2200 and 2300 m was not significant (**Table 1**). PCoA of the pairwise UniFrac distances for the bacterial communities in each sample indicated that bacterial phylogenetic structure tended to be relatively similar among samples within the same elevation and distinctly different among the different elevations (Supplementary Figure S1).

**Table 1:** Dissimilarities in bacterial OTU community composition between elevations on Changbai Mountain as determined by analysis of similarities (ANOSIM) *R*-values.

Elevation (m)	2100	2200	2300	2400	2500
2000	0.17	0.11	**0.96**	**0.48**	**1**
2100		0.23	**0.95**	**0.63**	**1**
2200			**0.5**	0.21	**0.96**
2300				**0.28**	**0.93**
2400					**0.68**

*An R-value near +1 means that there is dissimilarity between the groups, while an R-value near 0 indicates no significant dissimilarity between the groups. Values in bold indicate significant dissimilarity (*P* < 0.05).*

**FIGURE 1:**
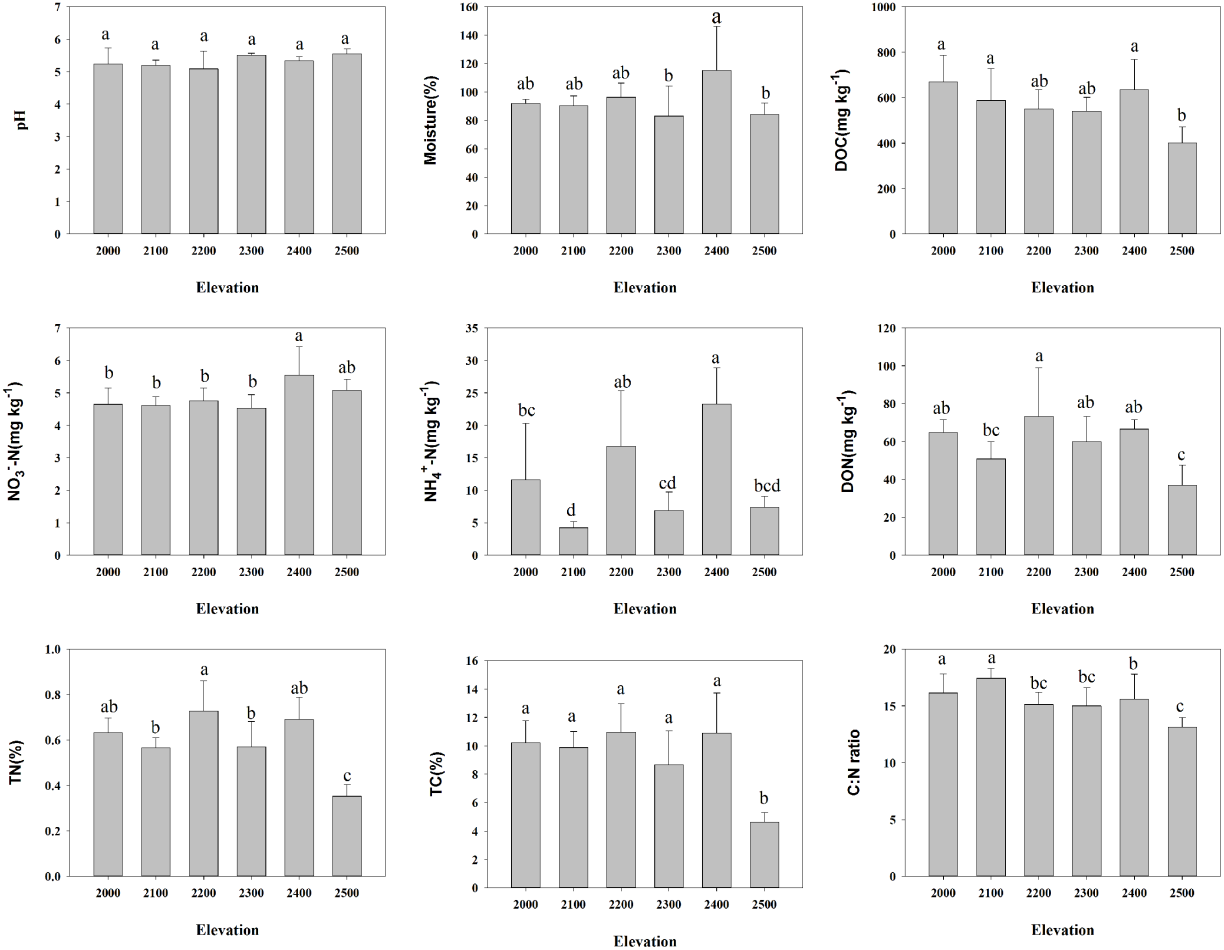
**Relationships between soil characteristics and elevation**. Error bars denote SD; different letters represent significant differences from Duncan comparisons (*P* < 0.05).

**FIGURE 2:**
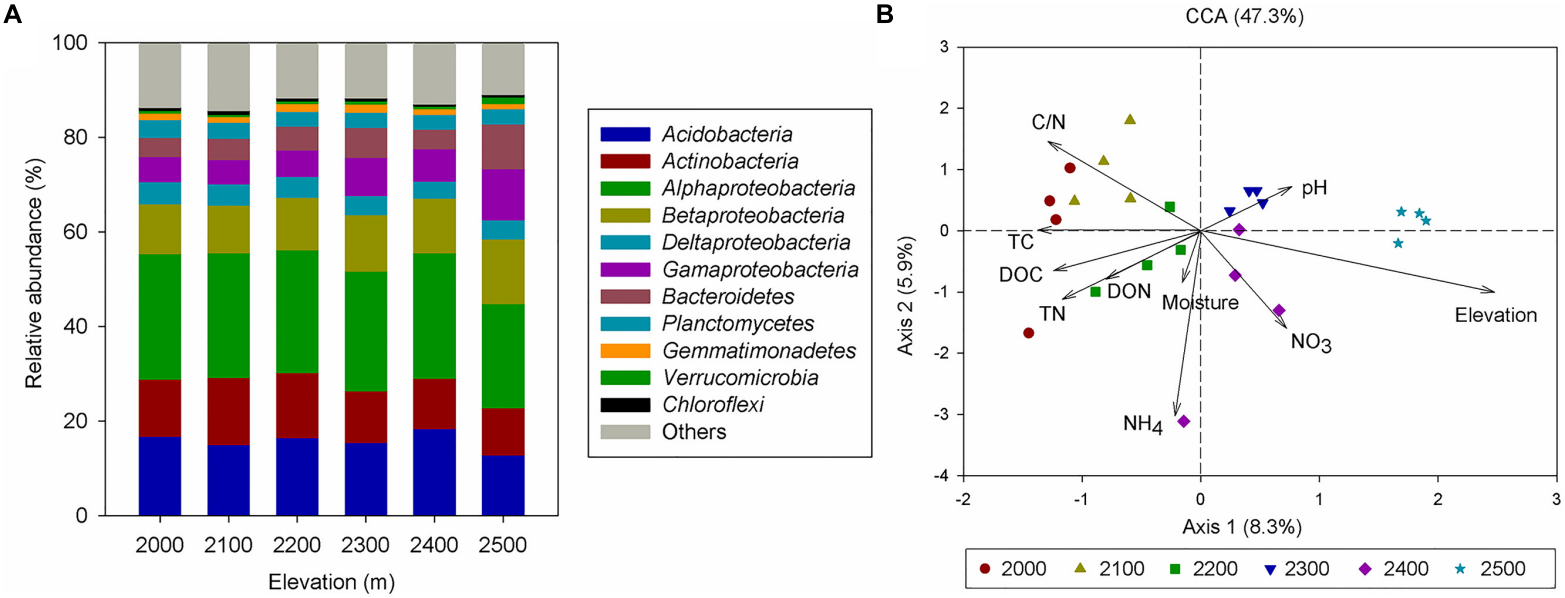
**(A)** Relative abundances of the dominant bacterial phyla in soils separated according to elevation categories. Relative abundances are based on the proportional frequencies of those DNA sequences that could be classified at the phylum level. (This figure is based on the information provided in Supplementary Table S4.) **(B)** Canonical correspondence analysis (CCA) of the bacterial communities with symbols coded by elevation category.

**FIGURE 3:**
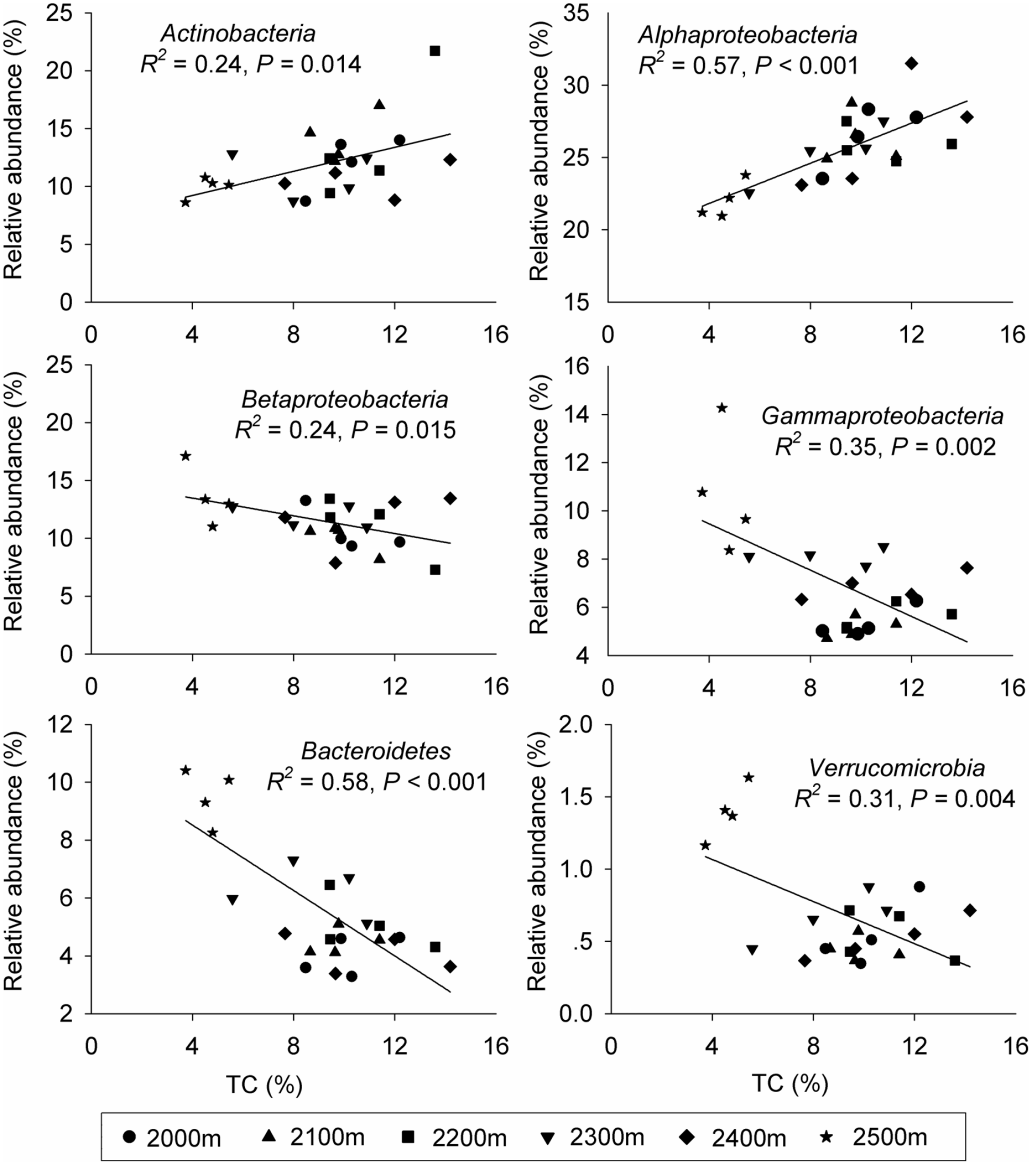
**The relative abundances of the dominant bacterial phyla at each elevation in relation to soil total carbon (TC)**. The strength of each relationship given is based on the linear regression equation.

Canonical correspondence analysis showed that elevation had the strongest effect (longer arrow) on bacterial community composition (**Figure 2B**). Of all the environmental variables tested, elevation was the most highly correlated with community composition (*r* = 0.64, *P* = 0.001, **Table 2**). These results suggest that elevation could be a good predictor of variation in bacterial community composition. Other factors such as TC, TN, C:N ratio, and DOC, also showed a high correlation with bacterial community composition based on Mantel test (**Table 2**). Specifically, significant relationships were found between the relative abundance of each taxonomic group and soil carbon and nitrogen contents (Supplementary Table S5). For example, the relative abundance of *Alphaproteobacteria*, *Actinobacteria* increased with TC, whereas the relative abundance of *Betaproteobacteria*, *Gammaproteobacteria*, and *Bacteroidetes* showed the opposite pattern. Even *Verrucomicrobia*, which had relatively low abundances, were significantly correlated with TC (**Figure 3**). Surprisingly, the relative abundance of *Acidobacteria* was significantly correlated with soil pH, despite the narrow pH ranges in these soils (**Figure 1**; Supplementary Table S5).

**Table 2:** Mantel test results for the correlation between community composition and environmental variables for bacteria along the elevational gradient.

Variable	*r*	*P*
Elevation	**0.63**	**0.001**
TC	**0.439**	**0.001**
TN	**0.423**	**0.001**
C:N ratio	**0.274**	**0.002**
DOC	**0.254**	**0.007**
DON	0.206	0.068
NH_4_^+^-N	0.16	0.063
NO_3_^-^-N	0.133	0.124
pH	0.098	0.201
Moisture	0.066	0.291

*TC, total carbon; TN, total nitrogen; DOC, dissolved organic carbon; DON, dissolved organic nitrogen. Values in bold indicate significant correlation (*P* < 0.05).*

### Phylotype Richness, Phylogenetic Diversity, Phylogenetic Signals, and Phylogenetic Relatedness for Bacterial Communities

**FIGURE 4:**
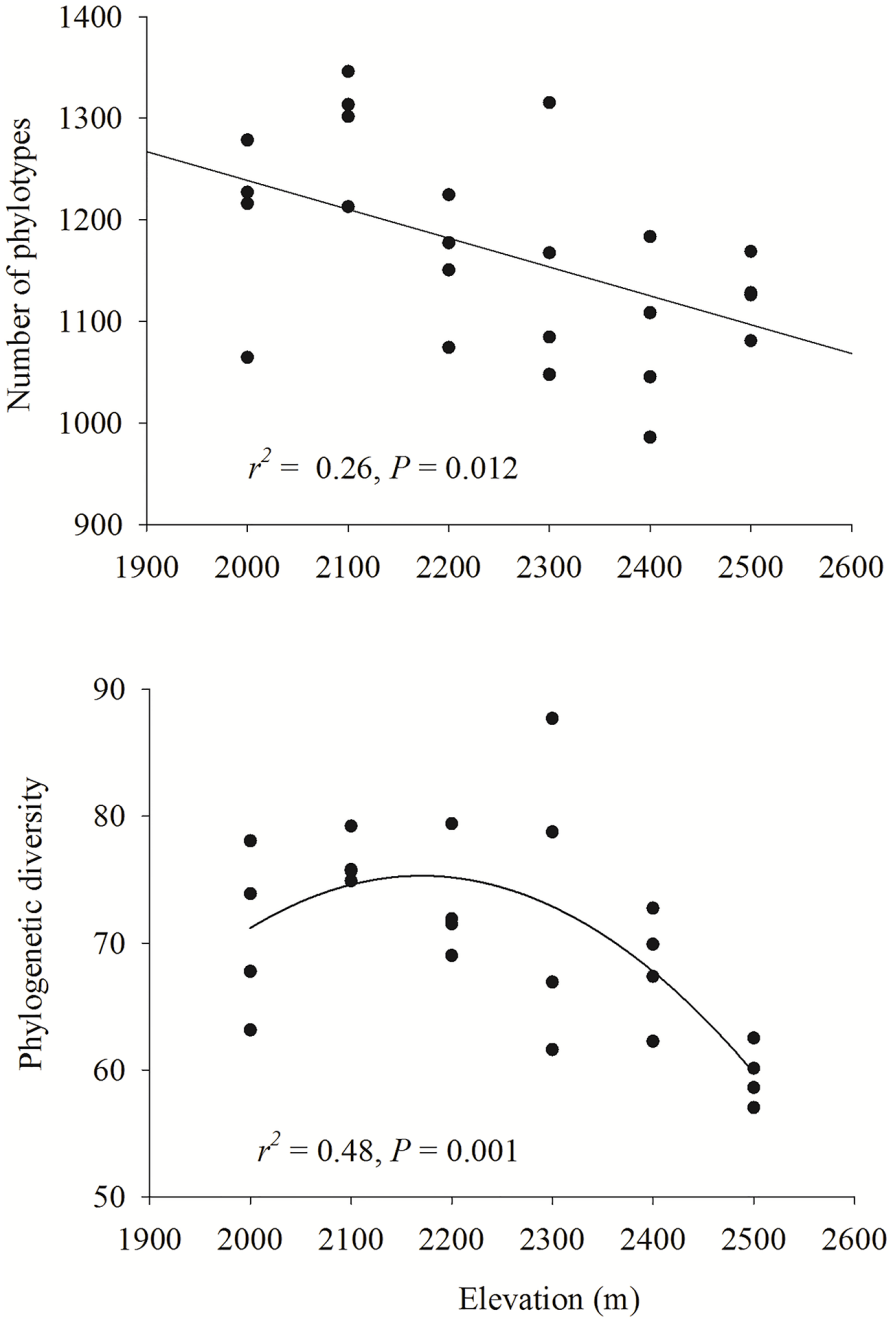
**Relationships between elevation and bacterial phylotype richness and phylogenetic diversity**. Linear or quadratic models were selected to describe the relationship. The communities were randomly sampled to obtain 4900 sequences.

Phylotype richness decreased with increased elevation (*r*^2^ = 0.26, *P* = 0.012), whereas Faith’s (1992) phylogenetic diversity exhibited a unimodal pattern with elevation (*r*^2^ = 0.48, *P* < 0.001; **Figure 4**).

**FIGURE 5:**
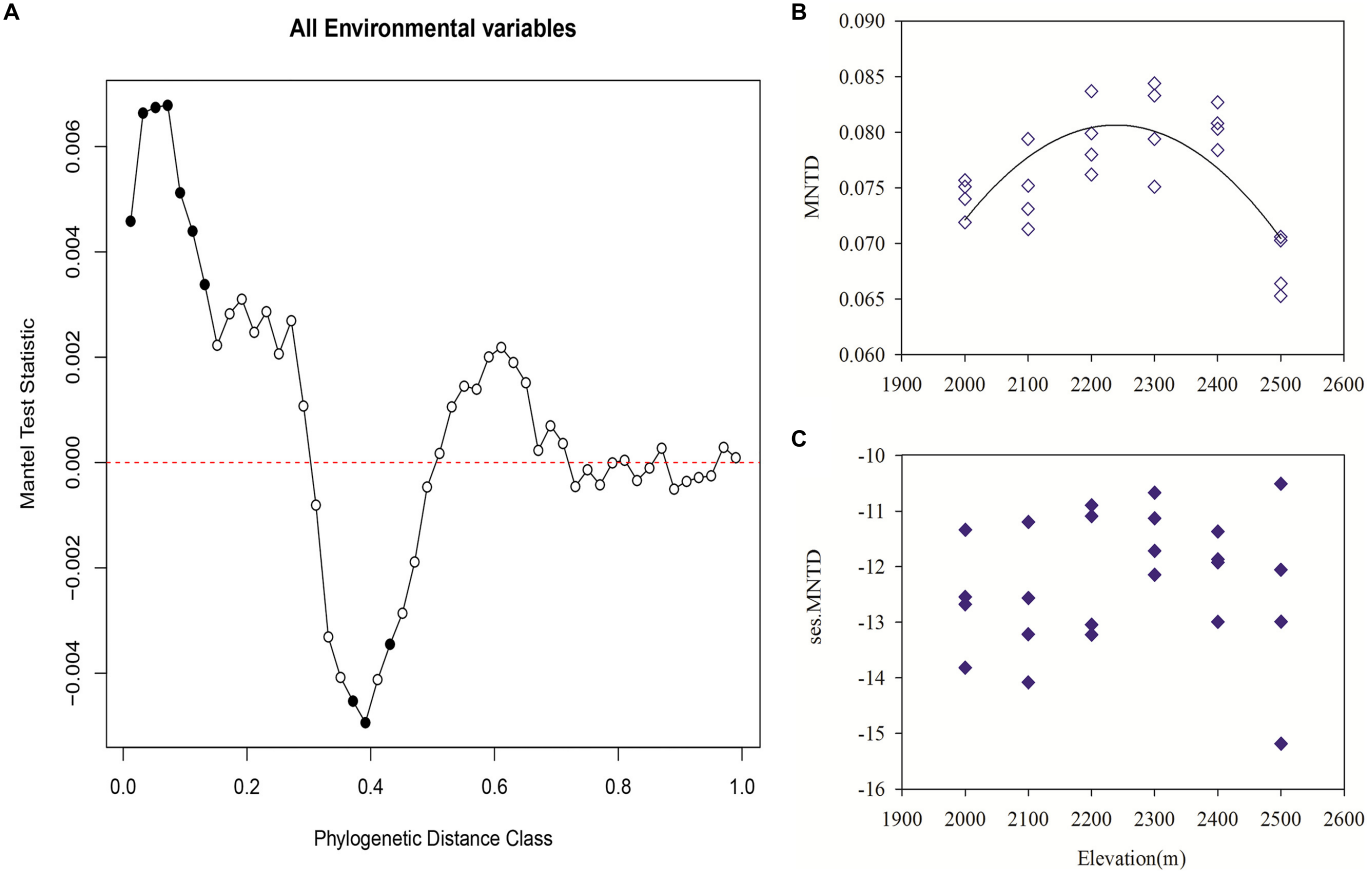
**Pearson correlation resulting from Mantel correlogram between the pairwise matrix of OTU niche distances and phylogenetic distances (with Jukes–Cantor model) for each sample group with 999 permutations (A)**. Significant correlations (*P* < 0.05, solid circles) indicate phylogenetic signal in species ecological niches **(A)**. Variation in community phylogenetic relatedness along the elevation gradient as measured with observed mean nearest taxon distance (MNTD; **B**) and the standardized effect sizes of MNTD **(C)**. MNTD followed a unimodal pattern with elevation (*r*^2^ = 0.53, *P* < 0.001). Significant ses.MNTD values were indicated as solid circles (*P* < 0.001, 1000 null model runs).

Mantel correlograms showed significant positive correlations across short phylogenetic distances. Meanwhile, there were significant negative correlations at intermediate phylogenetic distances and non-significant relationships across longer phylogenetic distances (**Figure 5A**). These results indicate that at short phylogenetic distances closely related bacterial taxa are phylogenetically conserved in their niches.

The MNTD showed that phylogenetic relatedness was closer among samples in lower or higher elevations than that in mid-elevation, which followed a unimodal pattern with elevation (*r*^2^ = 0.53, *P* < 0.001; **Figure 5B**). All of the standardized effect sizes of MNTD (ses.MNTD) that were obtained using the null model were significantly negative, which indicated that the bacterial communities had a tendency to be more phylogenetically clustered than expected by chance. However, the standardized metric did not show an apparent trend with elevation that was different from that of MNTD, which indicated that the generated random effects greatly impacted the elevational pattern of the phylogenetic structure (**Figure 5C**).

The correlations between biodiversity and environmental variables were examined by multiple OLS regression test. TC had the highest correlations with phylogenetic diversity and MNTD, and C:N ratio showed the highest correlation with OTU richness (**Table 3**).

**Table 3:** Relationships between bacterial diversity and potential explanatory variables that were modeled using multiple ordinary least squares regression.

	*r^2^*	AIC	Explanatory variables and β-weights				
OTU richness	0.461	-7.85	C:N ratio**	Moisture**	pH		
			0.557^a^	-0.685	-0.297		
Phylogenetic diversity	0.361	-3.78	TC**	Moisture**	DON		
			1.121	-0.759	-0.469		
MNTD	0.481	-4.76	TC**	Moisture*	NH_4_^+^-N*	DON*	pH
			1.503	-0.866	0.753	0.795	0.389

The best models were identified using Akaike’s information criterion (AIC). All of the variables were displayed with increasing P-values. TC, total carbon; DON, dissolved organic nitrogen. *P < 0.05, **P < 0.01.

*^a^Standardized partial regression coefficients.*

## Discussion

### Elevational Diversity Patterns

Our results showed that elevation strongly influenced the diversity of soil bacterial communities. Taxonomic richness linearly decreased with increased elevation and phylogenetic diversity exhibited a unimodal pattern with elevation. This observation was beyond our expectations, because a few studies have found elevational trends in soil bacterial diversity (Bryant et al., 2008; Singh et al., 2012, 2014), with most studies finding non-significant elevational patterns (Zhang et al., 2009; Fierer et al., 2011; Shen et al., 2013; Xu et al., 2014; Yuan et al., 2014). The pattern of decreasing richness might be caused by decreasing TC and DOC content with elevation (Supplementary Table S2). Our multiple OLS regression analyses revealed that TC and C:N ratio had strong correlations with taxonomic richness and diversity. In addition, we found the relative abundance of six dominant taxa had a significant correlation with elevation, as well as *Verrucomicrobia.* The relationships between these individual groups and elevation are likely contributing to the overall bacterial elevational pattern. Furthermore, significant correlations were also found between the relative abundance of these phyla with soil TC, TN, DOC, and DON, except for *Deltaproteobacteria*. These results suggest that soil carbon and nitrogen contents could be prominent contributors to the observed diversity patterns. Despite that, we cannot exclude other factors influencing this decreasing richness pattern. For example, soil moisture also has close correlations with bacterial richness and diversity based on OLS regression analyses in our study. Actually, several researches have revealed that moisture could be a controlling variable influencing bacterial diversity (Angel et al., 2010; Yuan et al., 2014; Zhang et al., 2014). It should be noted that the richness in 2000 and 2100 m elevations where the treeline formed by birch distributes was relatively higher than that in higher elevations. Recently, Thébault et al. (2014) studied microbial diversity at the treeline, which open new avenues for novel research. While dispersal ability is often claimed to be the main determinant of a species’ range (Brown et al., 1996; Lester et al., 2007), we speculate that species dispersal from forests to tundra soils might increase microbial diversity at lower elevations within alpine tundra. Soil pH, usually the best predictor of variation in microbial diversity, and might also influence elevational diversity patterns (Bryant et al., 2008). As expected, no significant correlation between pH and diversity was found in our study, which is largely due to the narrow pH range and low variance in pH among elevations. Although soil temperature was not measured here, given that air temperature is relatively constant along this elevational gradient, it is unlikely that the role of soil temperature would overwhelm that of soil carbon and nitrogen contents. In addition, although differences in sampling timing could lead to seasonal changes in bacterial diversity (Lipson and Schmidt, 2004; Björk et al., 2008), researchers have found that the spatial variations are stronger than the seasonal variations in alpine tundra (Zinger et al., 2009). Although taxonomic and phylogenetic diversity are typically related (as is the case in our study), the pattern with elevation showed that they might be slightly different (Morlon et al., 2011; Singh et al., 2012). For phylogenetic diversity, the unimodal pattern mainly resulted from the higher richness at 2300 m. To our knowledge, this is the first observation of significant elevation trend across such a small-scale elevational gradient. Clearly, the generality of these observations for microbes still needs to be addressed by more extensive studies for specific habitats, as well as across habitats.

### Phylogenetic Relatedness with Elevation

The influence of evolutionary history and ecological processes on community assembly can be assessed by analyzing the phylogenetic structures of communities. However, inferring ecological processes using phylogenetic information requires quantification of phylogenetic signal (Losos, 2008) in ecological niches (Cavender-Bares et al., 2009). Many studies on bacteria have found a positive relationship between phylogenetic distances and ecological differences among close relatives, which indicated that closely related bacterial taxa are ecologically coherent (Newton et al., 2007; Stegen et al., 2012; Wang et al., 2013). Our Mantel correlogram analyses showed that there were significant phylogenetic signals across short phylogenetic distances, consistent with the results of Stegen et al. (2012) and Wang et al. (2013). Our results combined with previous studies, indicate that at short phylogenetic distances closely related bacterial taxa are phylogenetically conserved in their niches. Since metrics of nearest taxon distances (for example, MNTD) focus on relatively short phylogenetic distance, the strong phylogenetic signal across short phylogenetic distances also suggests that these metrics are particularly suitable for ecological inferences.

In our study, the bacterial communities were more likely to show phylogenetic clustering than expected by chance at all elevations based on the standardized MNTD metric. This observation is consistent with reports for bacterial phylogenetic structures in the Chinese Laojun Mountain and Colorado Rocky Mountains (Bryant et al., 2008; Wang et al., 2012). However, the ses.MNTD did not show a significant trend with elevation, in contrast with Wang et al. (2012) who observed increasing phylogenetic clustering with elevation for biofilm bacteria in a stony stream. The contradictory results might caused by the differences between soil and aquatic environments. Similar to our results, Bryant et al. (2008) found a weak, non-significant increase in phylogenetic structure toward higher elevation for the relative abundance of soil *Acidobacteria*. One possible reason could be that the elevational gradient considered here (2000–2500 m) and in Bryant (2460–3380 m) might not have been large enough to delineate clear patterns. Interestingly, there is also not a consistent trend in phylogenetic structure with elevation for plants and animals based on previous studies. For example, an increasing phylogenetic over-dispersion for angiosperms with increased elevation has been shown in the Colorado Rocky Mountains, whereas Hoiss et al. (2012) found increasing phylogenetic clustering in bee communities with increased elevation.

The net relatedness index (NRI) and the nearest taxon index (NTI) were considered classical measures of phylogenetic relatedness and extensively used by researchers (ses.MNTD is equivalent to -1 times the NTI, Webb, 2000; Kembel and Hubbell, 2006; Bryant et al., 2008; Kembel, 2009). It should be noted that the elevation trend shown by ses.MNTD is different from the MNTD (non-significant trend vs. unimodal trend) in this study. Actually, the choice of an appropriate null model to use when measuring the structure of ecological communities has been very contentious (Gotelli, 2001; Kembel and Hubbell, 2006), and the debate has mainly focused on the relative merits of null models that maintain or do not maintain species frequencies (Gotelli and Graves, 1996). Importantly, the observed phylogenetic clustering here is based on the view that bacterial niches (the particular set of resources and environmental conditions that an individual species exploits, Prosser et al., 2007) are phylogenetically conserved, which has been demonstrated by our phylogenetic signal result. For the unimodal trend shown by MNTD with elevation, this result was similar to the trend of phylogenetic diversity with elevation. We interpret the lower phylogenetic relatedness in mid-elevation communities as a possible reason that the environmental filtering effect of abiotic factors is replaced here by an increased competition between species that was generated through evolutionary processes.

Despite the bias of different null models, we found the pattern that bacterial communities are more phylogenetically clustered at higher elevations, and this pattern has been observed in a number of studies not only for plants and animals (Graham et al., 2009; Kluge and Kessler, 2010; Machac et al., 2011) but also for microbes (Bryant et al., 2008; Wang et al., 2012). Helmus et al. (2010) suggests that ecosystem disturbances can result in assemblages that share many closely related species. There is also an evidence to show that environmental instability facilitates phylogenetic clustering for bacterial communities (Amaral-Zettler et al., 2011). Here, since TC (significantly correlated with TN, *R*^2^ = 0.88) was the strongest environmental filter for phylogenetic structure (MNTD) based on OLS regression analyses, we infer that the phylogenetic clustering at 2500 m elevation might be closely related to sharply low soil carbon and nitrogen contents. Taken together, despite the effect of species interactions and evolutionary processes, the results support our hypothesis, suggesting that environmental filtering process tend to be more prominent forces in structuring communities along elevation.

### Factors Influencing Bacterial Community Composition

Understanding the factors controlling bacterial community composition is a fundamental goal in microbial ecology (Martiny et al., 2006; Hanson et al., 2012). In this study, we found clear and significant differences in soil bacterial community composition among elevations (PCoA, CCA, ANOSIM analyses). Multivariate analyses also demonstrated that elevation had the highest correlation with bacterial community composition. This is surprising considering the small-scale elevational gradient studied here. Many studies of elevational gradient have documented strong differences in taxonomic community composition among elevations for bacteria (Singh et al., 2012, 2014; Wang et al., 2012; Shen et al., 2013) as well as for eukaryotic groups (Shen et al., 2014). Even for functional community composition, Yang et al. (2014) observed that the functional structure of the microbial community significantly differed among elevations. These results suggest that elevation could be a good predictor of variation in microbial community composition. Yet elevation is a complex and indirect gradient along which many environmental variables are changing. Soil pH has been widely recognized as a primary driver for soil bacterial horizontal distribution (Fierer and Jackson, 2006; Lauber et al., 2009; Chu et al., 2010; Griffiths et al., 2011). Recently, the importance of pH in structuring bacterial and eukaryotic microbial community composition was also found in elevational distributions (Yuan et al., 2014; Zhang et al., 2013; Shen et al., 2014). A significant pH effect was not detectable here, mainly because of the narrow pH range and limited variation among sampling sites. In contrast, our results showed that the composition of the whole bacterial community and the relative abundance of dominant phyla was closely correlated with soil TC, TN, DOC, DON, and C:N ratio. Such significant correlation between soil carbon and nitrogen contents and bacterial community structure has rarely been reported in studies of elevational gradient. For instance, Yuan et al. (2014) observed that precipitation and soil NH_4_^+^ were dominant environmental factors that influenced bacterial communities at 0–5 cm depth along the elevational gradients on the Tibetan Plateau. Other studies have found a significant relationship between soil C:N ratio and microbial community structure, but the effect was overwhelmed by pH (Shen et al., 2013; Zhang et al., 2013). It is well known that plants interact with soil microbial community through litter inputs and root exudates (Knelman et al., 2012). Recent advances in plant–microbe interactions research revealed that different plant species host specific microbial communities, suggesting a potential role of plant species shaping rhizosphere microbiome (Berendsen et al., 2012; Oh et al., 2012). Vegetation type has been often observed to significantly influence the microbial communities in forest or tundra soils (Wallenstein et al., 2007; Nielsen et al., 2010; Chu et al., 2011; Shen et al., 2013). However and indeed, soil bacteria are more sensitive to soil factors such as pH, moisture, organic matter content, and C:N ratio (Fierer and Jackson, 2006; Brockett et al., 2012; Liu et al., 2014). Given that plants can determine carbon and nitrogen source and alter soil physical and chemical environment (Wallenstein et al., 2007; Prescott and Grayston, 2013), plants indirectly affect soil microbial communities (Landesman et al., 2014). Nielsen et al. (2010) also observed that at a landscape scale the composition of bacterial communities was not directly associated with plants, but selected by soil pH and C:N ratio. Furthermore, Shi et al. (2015) recently observed that bacterial community composition was strongly correlated with soil pH and moisture content among four vegetation types in Arctic tundra, while in soils with similar pH and moisture content, variables associated with nitrogen transformations were important determinants of bacterial community structure. Vegetation heterogeneities may have potential effects on soil microbial communities, however, due to our lack of vegetation data, we cannot test the correlations between plant communities and soil bacterial communities. Although this study was not designed to directly examine the effect of tundra vegetation type on bacterial communities, we cannot exclude the possibility that plant communities indirectly influence bacterial community composition through alteration of soil carbon and nitrogen contents. Nevertheless, our results indicate that soil carbon and nitrogen contents were the dominant environmental factors determining bacterial community composition, and further suggest that niche-based environmental filtering processes strongly structured bacterial communities along this elevational gradient.

## Conclusion

In summary, we showed that soil bacterial communities in Changbai Mountain tundra differed with elevation and bacterial taxonomic richness significantly decreased with increasing elevation. Soil carbon and nitrogen contents were significantly correlated with bacterial diversity and community composition, as well as specific phyla. To the best of our knowledge, this is the first study to reveal significant diversity patterns across a small-scale elevational gradient and a significant effect of soil carbon and nitrogen contents but not pH in predicting the elevational distribution of soil bacterial communities. Analyses of phylogenetic relatedness revealed that niche-based environmental filtering processes (soil carbon and nitrogen here) played a critical role in structuring bacterial communities and shaping biodiversity patterns. Further work is needed to link biodiversity patterns with community phylogenetic structures to more fully understand the underlying mechanisms and relative importance of evolutionary and ecological processes.

## Conflict of Interest Statement

The authors declare that the research was conducted in the absence of any commercial or financial relationships that could be construed as a potential conflict of interest.

## Footnotes

1 http://www.mothur.org/wiki/454_SOP

2 http://www.mothur.org/wiki/Pre.cluster

3 http://metagenomics.anl.gov/

4 http://www.r-project.org
