# Supplementary material for: Distinct soil bacterial communities along a small-scale elevational gradient in alpine tundra
Source: Front Microbiol. 2015 Jun 9;6:582. doi: 10.3389/fmicb.2015.00582 (PMC4493907; doi:10.3389/fmicb.2015.00582)

Table S1: Summary of geographic coordinates of sampling site.

| Site number | Elevation (m) | Latitude (N) | Longitude (E) |
| --- | --- | --- | --- |
| 1 | 2000 | 42°03′17″ | 128°04′02″ |
| 2 | 2000 | 42°03′18″ | 128°04′02″ |
| 3 | 2000 | 42°03′19″ | 128°04′01″ |
| 4 | 2000 | 42°03′21″ | 128°04′00″ |
| 5 | 2100 | 42°03′11″ | 128°03′55″ |
| 6 | 2100 | 42°03′10″ | 128°03′56″ |
| 7 | 2100 | 42°03′10″ | 128°03′57″ |
| 8 | 2100 | 42°03′13″ | 128°03′54″ |
| 9 | 2200 | 42°02′51″ | 128°03′56″ |
| 10 | 2200 | 42°02′52″ | 128°03′55″ |
| 11 | 2200 | 42°02′53″ | 128°03′53″ |
| 12 | 2200 | 42°02′58″ | 128°03′51″ |
| 13 | 2300 | 42°02′23″ | 128°03′56″ |
| 14 | 2300 | 42°02′24″ | 128°03′58″ |
| 15 | 2300 | 42°02′25″ | 128°03′59″ |
| 16 | 2300 | 42°02′25″ | 128°04′02″ |
| 17 | 2400 | 42°02′10″ | 128°04′00″ |
| 18 | 2400 | 42°02′12″ | 128°03′59″ |
| 19 | 2400 | 42°02′12″ | 128°03′57″ |
| 20 | 2400 | 42°02′11″ | 128°03′56″ |
| 21 | 2500 | 42°02′02″ | 128°03′55″ |
| 22 | 2500 | 42°02′02″ | 128°03′53″ |
| 23 | 2500 | 42°02′02″ | 128°03′54″ |
| 24 | 2500 | 42°02′03″ | 128°03′55″ |

Table S2: Tundra soil characteristics on Changbai Mountain. SM: soil moisture; DOC: dissolved organic carbon; DON: dissolved organic nitrogen; TN: total nitrogen; TC: total carbon.

| Elevation (m) | pH | SM (%) | DOC (mg/kg) | DON (mg/kg) | NO_3_^-^-N (mg/kg) | NH_4_^+^-N (mg/kg) | TN (%) | TC (%) | C:N ratio |
| --- | --- | --- | --- | --- | --- | --- | --- | --- | --- |
| 2000 | 5.72 | 92.5 | 835 | 61.4 | 4.4 | 5.6 | 0.56 | 9.88 | 17.6 |
| 2000 | 5.23 | 87.6 | 659 | 69.4 | 4.3 | 9.0 | 0.67 | 10.30 | 15.4 |
| 2000 | 5.44 | 94.5 | 609 | 71.2 | 4.5 | 7.4 | 0.70 | 12.20 | 17.4 |
| 2000 | 4.57 | 92.8 | 575 | 57.3 | 5.4 | 24.5 | 0.60 | 8.48 | 14.1 |
| 2100 | 5.01 | 100.0 | 492 | 62.6 | 4.8 | 4.4 | 0.63 | 11.40 | 18.1 |
| 2100 | 5.13 | 88.5 | 790 | 53.3 | 4.9 | 5.3 | 0.56 | 9.64 | 17.2 |
| 2100 | 5.42 | 89.4 | 580 | 44.5 | 4.4 | 3.0 | 0.53 | 8.66 | 16.3 |
| 2100 | 5.18 | 83.5 | 486 | 43.3 | 4.4 | 4.2 | 0.54 | 9.78 | 18.1 |
| 2200 | 5.29 | 104.8 | 502 | 67.6 | 4.5 | 17.0 | 0.74 | 11.40 | 15.4 |
| 2200 | 4.28 | 91.2 | 461 | 67.3 | 4.8 | 22.8 | 0.69 | 9.45 | 13.7 |
| 2200 | 5.26 | 104.4 | 648 | 109.4 | 5.3 | 22.7 | 0.90 | 13.60 | 15.1 |
| 2200 | 5.51 | 84.6 | 591 | 48.9 | 4.4 | 4.6 | 0.58 | 9.43 | 16.3 |
| 2300 | 5.53 | 99.2 | 600 | 60.2 | 4.8 | 8.2 | 0.63 | 10.20 | 16.2 |
| 2300 | 5.51 | 56.1 | 465 | 47.5 | 3.9 | 3.4 | 0.44 | 5.58 | 12.7 |
| 2300 | 5.56 | 100.4 | 581 | 78.6 | 4.8 | 10.0 | 0.69 | 10.9 | 15.8 |
| 2300 | 5.4 | 76.4 | 513 | 53.4 | 4.6 | 6.0 | 0.52 | 7.99 | 15.4 |
| 2400 | 5.44 | 100.8 | 741 | 62.5 | 5.2 | 30.1 | 0.70 | 9.66 | 13.8 |
| 2400 | 5.14 | 78.4 | 439 | 63.5 | 4.5 | 22.1 | 0.55 | 7.66 | 13.9 |
| 2400 | 5.41 | 136.1 | 675 | 73.2 | 6.4 | 24.3 | 0.74 | 12.00 | 16.2 |
| 2400 | 5.34 | 145.1 | 684 | 67.2 | 6.1 | 16.7 | 0.77 | 14.20 | 18.4 |
| 2500 | 5.60 | 78.0 | 374 | 37.2 | 4.6 | 8.2 | 0.38 | 4.51 | 11.9 |
| 2500 | 5.64 | 79.2 | 373 | 28.8 | 5.0 | 6.1 | 0.28 | 3.73 | 13.3 |
| 2500 | 5.32 | 95.7 | 505 | 51.9 | 5.5 | 9.4 | 0.40 | 5.44 | 13.6 |
| 2500 | 5.63 | 84.4 | 352 | 29.7 | 5.2 | 5.9 | 0.35 | 4.80 | 13.7 |

Table S3: Number of sequence reads (before (BF) and after (AF) quality filtering using Mothur software) and OTUs classified from after quality filtering sequences.

| Site number | Elevation(m) | BF Sequences | AF Sequences | OTUs |
| --- | --- | --- | --- | --- |
| 1 | 2000 | 10711 | 9684 | 1828 |
| 2 | 2000 | 11964 | 11220 | 1692 |
| 3 | 2000 | 15170 | 14125 | 2236 |
| 4 | 2000 | 10560 | 9676 | 1924 |
| 5 | 2100 | 14112 | 13179 | 2410 |
| 6 | 2100 | 11483 | 10832 | 2078 |
| 7 | 2100 | 10176 | 9316 | 1914 |
| 8 | 2100 | 8740 | 8059 | 1608 |
| 9 | 2200 | 8702 | 8090 | 1408 |
| 10 | 2200 | 11376 | 10124 | 1797 |
| 11 | 2200 | 8628 | 7838 | 1497 |
| 12 | 2200 | 7249 | 6615 | 1458 |
| 13 | 2300 | 6087 | 5521 | 1249 |
| 14 | 2300 | 5597 | 4931 | 1320 |
| 15 | 2300 | 12602 | 11530 | 1674 |
| 16 | 2300 | 10523 | 9719 | 1588 |
| 17 | 2400 | 9389 | 8715 | 1346 |
| 18 | 2400 | 9522 | 9001 | 1651 |
| 19 | 2400 | 8957 | 8181 | 1481 |
| 20 | 2400 | 8252 | 7600 | 1326 |
| 21 | 2500 | 23492 | 20477 | 2489 |
| 22 | 2500 | 20733 | 18098 | 2373 |
| 23 | 2500 | 21271 | 18142 | 2193 |
| 24 | 2500 | 18355 | 16556 | 2262 |

Table S4: Relative average abundances of phyla classified with RDP taxonomy across all soils and soils grouped into different elevation categories (values represent % of total non-redundant sequences). Asterisks indicate sequences classified to the domain Bacteria, but not to a specific phylum. The “0.00” value means less than 0.01.

| Phylum | All soils | 2000 m | 2100 m | 2200 m | 2300 m | 2400 m | 2500 m |
| --- | --- | --- | --- | --- | --- | --- | --- |
| *Alphaproteobacteria* | 26.35 | 28.03 | 27.72 | 26.72 | 26.96 | 27.49 | 23.77 |
| *Acidobacteria* | 17.16 | 18.73 | 16.65 | 18.20 | 16.75 | 20.75 | 14.71 |
| *Betaproteobacteria* | 11.33 | 9.43 | 8.92 | 10.56 | 11.71 | 11.20 | 13.91 |
| *Actinobacteria* | 10.70 | 11.38 | 13.41 | 12.86 | 10.07 | 9.67 | 8.68 |
| *Gammaproteobacteria* | 7.28 | 5.16 | 5.19 | 5.75 | 8.17 | 7.13 | 9.95 |
| *Bacteroidetes* | 5.94 | 4.10 | 4.36 | 4.84 | 5.96 | 4.23 | 9.04 |
| *Bacteria** | 4.53 | 5.02 | 4.77 | 4.83 | 4.62 | 3.28 | 4.48 |
| *Deltaproteobacteria* | 3.71 | 4.14 | 4.16 | 4.15 | 3.63 | 3.66 | 3.10 |
| *Chloroflexi* | 3.29 | 4.32 | 5.32 | 2.95 | 2.51 | 3.38 | 2.08 |
| *Planctomycetes* | 3.17 | 3.43 | 3.66 | 3.04 | 3.24 | 3.14 | 2.79 |
| *Proteobacteria** | 1.46 | 1.41 | 1.43 | 1.34 | 1.49 | 0.96 | 1.77 |
| *Gemmatimonadetes* | 1.13 | 1.22 | 1.17 | 1.30 | 1.42 | 1.23 | 0.81 |
| *Armatimonadetes* | 0.85 | 0.70 | 0.91 | 0.66 | 0.87 | 0.74 | 1.03 |
| *Verrucomicrobia* | 0.60 | 0.42 | 0.32 | 0.35 | 0.46 | 0.42 | 1.10 |
| *Elusimicrobia* | 0.53 | 0.64 | 0.46 | 0.64 | 0.54 | 0.63 | 0.40 |
| *Cyanobacteria* | 0.47 | 0.27 | 0.32 | 0.22 | 0.31 | 0.37 | 0.87 |
| *Firmicutes* | 0.25 | 0.48 | 0.25 | 0.45 | 0.08 | 0.21 | 0.13 |
| *TM7* | 0.21 | 0.17 | 0.19 | 0.26 | 0.21 | 0.19 | 0.24 |
| *AD3* | 0.18 | 0.19 | 0.16 | 0.13 | 0.18 | 0.35 | 0.14 |
| *OP3* | 0.18 | 0.10 | 0.07 | 0.11 | 0.19 | 0.21 | 0.29 |
| *WPS-2* | 0.16 | 0.17 | 0.17 | 0.12 | 0.19 | 0.24 | 0.14 |
| *Chlorobi* | 0.16 | 0.16 | 0.10 | 0.19 | 0.12 | 0.16 | 0.20 |
| *TM6* | 0.15 | 0.10 | 0.13 | 0.11 | 0.13 | 0.18 | 0.20 |
| *WS3* | 0.04 | 0.05 | 0.06 | 0.03 | 0.04 | 0.07 | 0.01 |
| *OD1* | 0.04 | 0.05 | 0.03 | 0.07 | 0.05 | 0.02 | 0.02 |
| *Fibrobacteres* | 0.03 | 0.03 | 0.01 | 0.06 | 0.02 | 0.02 | 0.04 |
| *OP11* | 0.02 | 0.01 | 0.01 | 0.01 | 0.02 | 0.02 | 0.03 |
| *BRC1* | 0.02 | 0.02 | 0.01 | 0.02 | 0.02 | 0.03 | 0.01 |
| *WYO* | 0.02 | 0.01 | 0.02 | 0.01 | 0.01 | 0.01 | 0.03 |
| *Nitrospirae* | 0.01 | 0.02 | 0.02 | 0.01 | 0.01 | 0.02 | 0.00 |
| *Fusobacteria* | 0.01 | 0.01 | 0.00 | 0.01 | 0.01 | 0.01 | 0.01 |
| *Chlamydiae* | 0.00 | 0.01 | 0.01 | 0.00 | 0.01 | 0.00 | 0.00 |
| *GN02* | 0.00 | 0.00 | 0.00 | 0.00 | 0.00 | 0.00 | 0.00 |
| *FCPU426* | 0.00 | 0.00 | 0.00 | 0.00 | 0.00 | 0.00 | 0.00 |
| *NKB19* | 0.00 | 0.00 | 0.00 | 0.00 | 0.00 | 0.00 | 0.00 |
| *OP9* | 0.00 | 0.00 | 0.00 | 0.00 | 0.00 | 0.00 | 0.00 |
| *Spirochaetes* | 0.00 | 0.00 | 0.00 | 0.00 | 0.00 | 0.00 | 0.00 |
| *SR1* | 0.00 | 0.00 | 0.00 | 0.00 | 0.00 | 0.00 | 0.00 |
| *Tenericutes* | 0.00 | 0.00 | 0.00 | 0.00 | 0.00 | 0.00 | 0.00 |
| *Thermi* | 0.00 | 0.00 | 0.00 | 0.00 | 0.00 | 0.00 | 0.00 |
| *WS2* | 0.00 | 0.00 | 0.00 | 0.00 | 0.00 | 0.00 | 0.00 |

Table S5: Pearson correlations (R) between the relative abundances of the main bacterial phyla and soil and site characteristics. Values in bold indicate significant correlations (*P* < 0.05).

| R | Elevation | pH | % Moisture | NO_3_^-^-N | NH_4_^+^-N | DOC | DON | % TN | % TC | C:N ratio |
| --- | --- | --- | --- | --- | --- | --- | --- | --- | --- | --- |
| *Acidobacteria* | -0.21 | **-0.56** | 0.16 | 0.08 | 0.68 | 0.2 | 0.28 | **0.46** | 0.3 | -0.06 |
| *Actinobacteria* | **-0.41** | 0.07 | 0.09 | -0.16 | -0.12 | 0.28 | **0.53** | **0.45** | **0.49** | 0.38 |
| *Alphaproteobacteria* | **-0.44** | -0.07 | **0.57** | 0.26 | 0.06 | **0.68** | **0.52** | **0.64** | **0.76** | **0.72** |
| *Betaproteobacteria* | **0.47** | 0.1 | -0.06 | 0.17 | -0.12 | **-0.41** | **-0.54** | **-0.52** | **-0.49** | -0.32 |
| *Deltaproteobacteria* | **-0.54** | -0.25 | -0.13 | -0.23 | -0.19 | -0.04 | -0.04 | 0.01 | 0.06 | 0.22 |
| *Gammaproteobacteria* | **0.77** | **0.45** | -0.17 | 0.11 | -0.15 | **-0.54** | **-0.41** | **-0.55** | **-0.59** | **-0.58** |
| *Bacteroidetes* | **0.66** | **0.41** | -0.37 | -0.02 | **-0.41** | **-0.64** | **-0.6** | **-0.77** | **-0.76** | **-0.51** |
| *Gemmatimonadetes* | -0.19 | 0.16 | -0.3 | **-0.49** | -0.06 | 0.01 | 0.39 | 0.27 | 0.21 | 0.05 |
| *Planctomycetes* | -0.38 | -0.15 | -0.07 | 0.05 | 0.01 | 0.39 | 0.15 | 0.06 | 0.04 | 0.07 |
| *Verrucomicrobia* | **0.63** | 0.39 | -0.08 | 0.18 | -0.29 | **-0.52** | **-0.49** | **-0.58** | **-0.56** | **-0.4** |
| *Chloroflexi* | -0.34 | -0.31 | **-0.42** | **-0.47** | -0.16 | -0.08 | -0.02 | -0.07 | -0.07 | 0.03 |

Figure S1: Principal coordinates analysis (PCoA) of unweighted UniFrac distances of the bacterial communities comparing all 24 samples from the six different elevations in Changbai Mountain tundra. Sites have been color-coded according to elevation gradient.


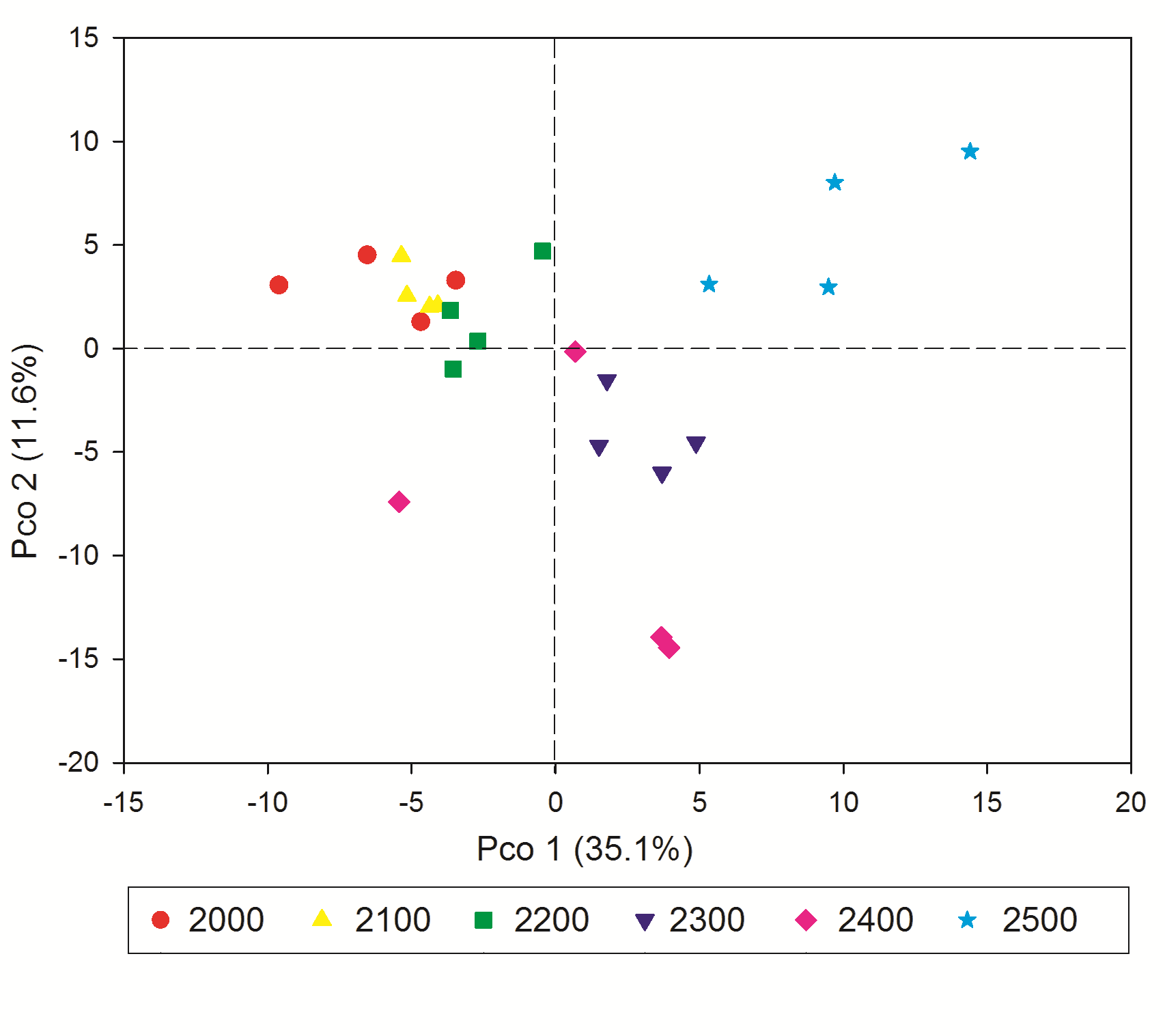


Figure S2: A view of the elevation transect in Changbai Mountain tundra.


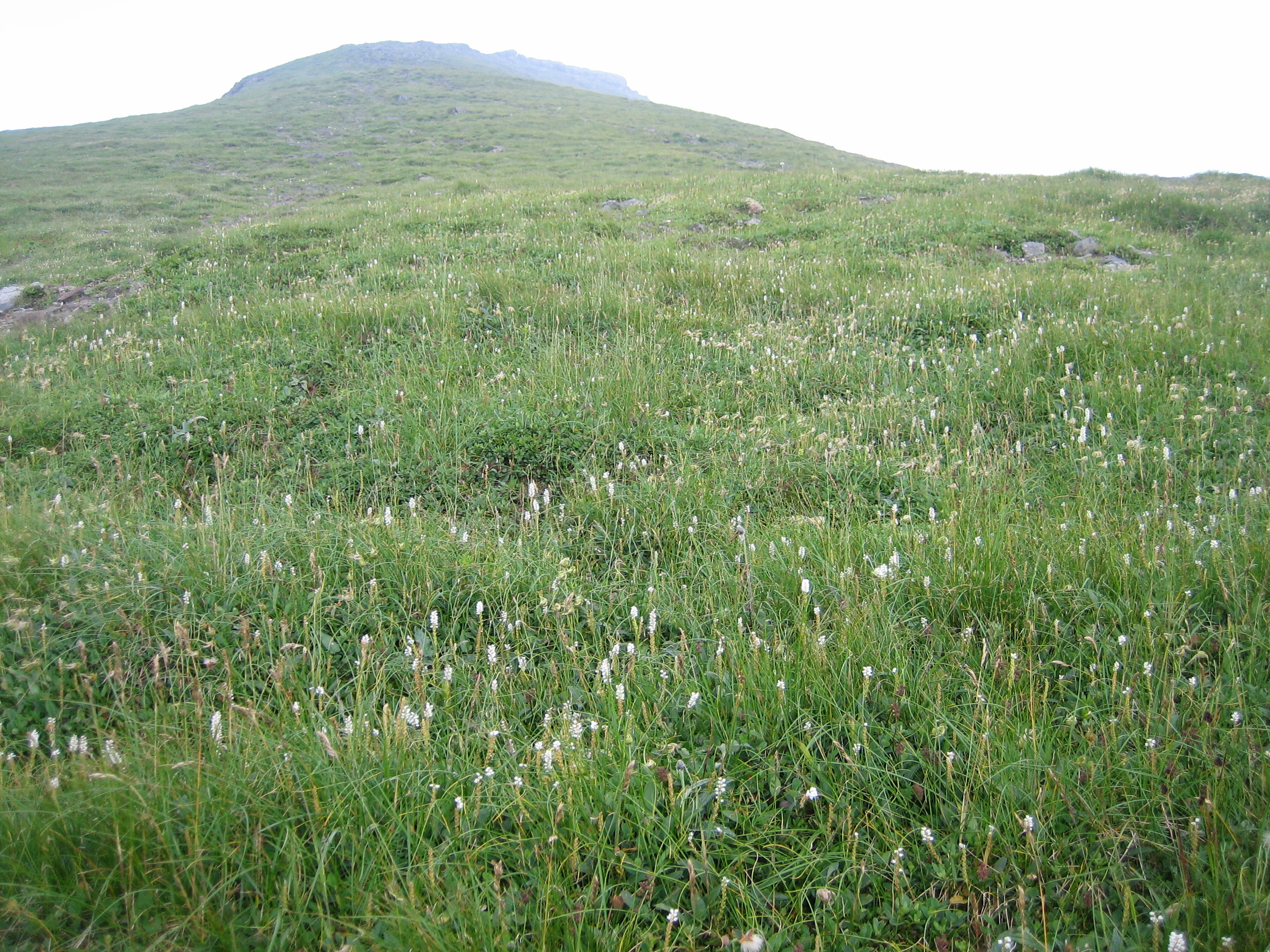

Supplement: Supplementary file 1 [file Data_Sheet_1.DOC]
